# Using Health Belief Model to Predict Hepatitis B Vaccination Uptake Among Undergraduate Nursing Students

**DOI:** 10.24248/eahrj.v8i2.790

**Published:** 2024-06-26

**Authors:** Gloria D Munuo, Golden Mwakibo Masika

**Affiliations:** a Department of Clinical Nursing, School of Nursing and Public Health, University of Dodoma; b Ministry of Health and Social Welfare

**Keywords:** Hepatitis B, Hepatitis B vaccine, Health Belief Model, Nursing students

## Abstract

**Background:**

Undergraduate nursing students in clinical practice have a higher risk of hepatitis B infection. The prevalence and factors associated with hepatitis B vaccination (HBV vaccine) uptake among nursing students remained unknown. This study examined the prevalence and factors associated with HBV vaccination among clinical nursing students.

**Methodology:**

A sample of 229 undergraduate nursing students was enrolled in an analytical cross-sectional study. Sociodemographic data, status of vaccination, and beliefs about HBV infection and vaccination using domains of the health belief model (HBM) were collected in a face-to-face interview using a questionnaire. Descriptive statistics were used to summarise the participants' characteristics and prevalence of HBV vaccination. Multivariate logistic regression analysis was used to examine the association between sociodemographic factors and domains of the HBM model and HBV vaccination uptake.

**Results:**

The prevalence of vaccination uptake was 25.8%. Sociodemographic factors associated with uptake of the HBV vaccine included being female (P =.031), being a final-year student (*P =.013*), and having knowledge of HBV (*P =.049*). As for HBM, two domains, perceived benefit [Adjusted Odds Ratio (AOR) = 1.40; 95% CI, 1.05 to 1.86; *P=.022*] and self-efficacy (AOR = 1.87, 95% CI, 1.12 to 3.11; *P=.016*), were significantly associated with HBV vaccine uptake.

**Conclusion:**

HBV vaccination uptake among undergraduate clinical nursing students was low. Clinical experience, knowledge, perceived benefit, and self-efficacy were positively associated with HBV vaccine uptake. Interventions to improve these domains among BSc Nursing students should be promoted to improve vaccination uptake.

## BACKGROUND

Hepatitis B is a serious, life-threatening infection of the liver caused by the Hepatitis B virus (HBV).^1,2^ It is a serious global health problem that affects around 296 million people worldwide, with 1.5 million new cases each year.^1^ The estimated global prevalence of HBV infection is 3.5% in the general population.^3^ This prevalence varies geographically, with 68% of the infected population found in Africa and the Western Pacific Regions. The prevalence is as low as 0.2% in higher-income countries like America and more than 3% to 8% in low- and middle-income countries like Sub-Saharan Africa and East Asia.^4,5^ In Tanzania, the burden of HBV infection is estimated at 4.3%; however, subpopulation studies in different parts of the country show that the prevalence could range from 3.5% to 20%.^6,7^

HBV can be transmitted through contact with body fluids such as blood, semen, or others from an infected person to a non-infected one. This can happen through unprotected sexual contact, needle stick injuries, transfusions, splashes, or from mother to child at birth.^2,8^ Studies show that anyone can be infected with HBV, but some groups of people, such as infants born to an infected mother, people who inject drugs, sex partners of people with hepatitis, men who have sex with men, haemodialysis patients, people who live with hepatitis patients, health care workers, and public safety workers exposed to blood on the job, are at higher risk.^8–12^ Unvaccinated populations are also at increased risk of infection when compared with other population groups.^11,13^ Due to work-related injuries the risk among healthcare workers could go fourfold higher than in other populations^14^ and the prevalence of about 7.4% to 11.8% of the infection has been observed among healthcare workers in Tanzania.^13,15^

If HBV infection progresses to chronic hepatitis, it can possibly lead to liver failure, cirrhosis, and hepatocellular carcinoma^16,17^ and ultimately lead to mortality. In 2019, a total of 820,000 deaths occurred due to the consequences of chronic hepatitis B infection.^1^ Screening and vaccination can be effective ways to reduce the risk of HBV infection among high-risk population groups. Nevertheless, studies have shown several barriers to screening for HBV and accepting to be vaccinated against HBV. Among these barriers are poor perceptions of vaccine safety, poor perceptions of the risk of infection, a lack of knowledge about HBV or the vaccine, misinformation, a lack of health insurance, a lack of familiarity with HBV guidelines, and poor knowledge of the risk of infection.^18,19^

**Figure 1.**
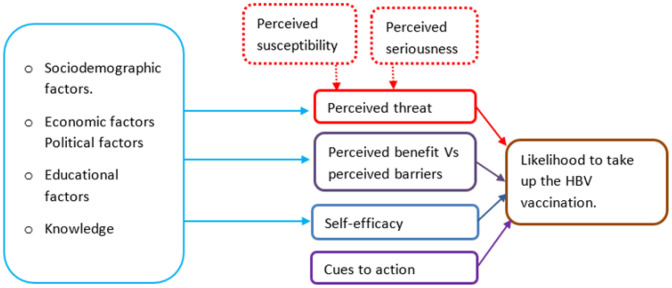
The Health Belief Model

Health professional students, including nursing students, are among the high-risk groups for contacting the disease, especially during the early stages of their clinical practice.^20^ A tool to map the variables that can predict their likelihood of taking the HBV vaccine is recommended for the proper planning of relevant interventions. The Health Belief Model (HBM) is hypothesised to be appropriate for this purpose. The HBM developed by Hochbaum, Rosenstock, & Kegels (1952), as cited by McKellar & Sillence^21^ attempts to explain and predict an intent to adopt health behaviors. The prediction of this intent is described through several theoretical constructs, such as perceived severity of a threat or disease, perceived susceptibility to that threat or disease, perceived benefits of taking appropriate health behaviours, perceived barriers, modifying variables, cues to action, and self-efficacy.^21^ Figure 1 illustrates the relationship between the constructs of HBM. In the context of HBV, the first two individual perceptions—perceived seriousness of HBV and perceived susceptibility of getting infected—defined the impending threat of HBV on the individual, whereas the excess of perceived benefit to take the vaccine minus perceived barriers are the main determinants as to whether a nursing student will take up HBV vaccination. The demographic variables and cues to action, such as the illness of a family member or classmate, practicing health facility emphasis or vaccination campaigns, accessibility of services in health facilities, cost-effectiveness of the vaccine, and national immunisation programmes, may modify one's perception of risk and benefit and moderate the variables that determine the likelihood of the behaviour to take up the HBV vaccination.^22^ In addition, self-efficacy, as a person's level of confidence in their ability to successfully perform a particular health behaviour, sums up the predictive power of the likelihood of taking up the HBV vaccination. To devise appropriate interventions for improving uptake of HBV screening and vaccination among health nursing students, it is crucial that factors that hinder them are studied and known. Therefore, the aim of this study was to examine the prevalence, uptake of HBV vaccine and association between nursing students' social demographic factors, domains of health belief model (HBM) and the uptake of HBV vaccination.

## METHODOLOGY

### Design and Settings

This study adopted an analytical, cross-sectional design. It was conducted among undergraduate nursing students at the University of Dodoma. Located in Dodoma City, the capital and centre of Tanzania, this is one of the large universities in Tanzania and in East Africa that host about 40,000 students.^24^ The School of Nursing and Public Health of this university hosts about 1200 undergraduate students in different programmes.

### Study Population

The study population involved undergraduate nursing students who had started their clinical practice; second to fourth years of study. Nursing students in their clinical practice years were practicing in six hospitals in the Dodoma and Singida regions. The inclusion criteria were being a student in the Bachelor of Science in Nursing programme from the University of Dodoma in the clinical year of study, thus having some baseline understanding of hepatitis viral infection from the Microbiology course, and being available for a face-to-face interview with the researcher. The exclusion criteria were being sick at the time of study, which may have interfered with one's ability to respond to the interview, or refusing to participate.

### Sample size calculation and selection

The sample was calculated using Cochran formula and then applying the formula for finite population.

where n=sample size, z=confidence level at 95% (1.96), p=proportion, in this study a p of 50% (0.5) was conservatively used as there was no reference study in with similar population characteristics; e = estimation error 5% (0.05) and n = sample size.



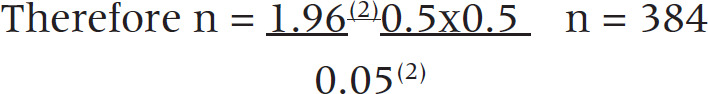



As the number of students in clinical practice as source population is less than 10000 then the formula for finite population was applied for calculating the finite sample.

whereas n’ =finite sample size, n = calculated sample size, = 384 and N = size of source population, = 568



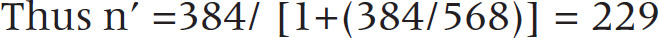



### Variable Measurements Dependent Variables

The dependent variable of this study was vaccination status-whether or not a nursing student has ever taken the HBV vaccine. This was determined by responding to two questions: whether or not the student had ever participated in screening for HBV infection and whether or not the student had ever been vaccinated for HBV. The responses were measured on a nominal scale with binary responses of “Yes” or “No”.

### Predictor Variables

Comprised of socio-demographic characteristics, knowledge of HBV, and domains of the HBM as they reflect factors that may influence the uptake of vaccination. Sociodemographic characteristics comprise age, sex, year of study, marital status, father's or male guardian's occupation, mother's or female guardian's occupation, source of financial support to meet daily needs, and type of insurance he or she uses. General knowledge of HBV, its transmission methods, risk factors for acquiring HBV, effects and complications of HBV, prevention, and treatment was measured using a set of 23 questions developed by the research team based on recommendations from previous literature ^25–27^ and pretested to a few medical students in clinical years. Components of HBM were measured using 11 Likert scale items on a scale of 1 to 5. The scale was developed and agreed upon by the research team based on interpretations of the literature about HBV and HBM.^21,22^ To attain face validity, all the instruments were pretested among 15 diploma-in-nursing students who have started clinical practice but are not part of the study population. The components of questionnaire comprised of perceived threat of HBV that was measured using 5 items having a score range of 5 to 25 points, perceived benefit of HBV vaccination measured using one item with a score range of 1 to 5 points, perceived barriers measured using 2 items with a score range of 2 to 10 points, cues to action measured using two items with a score range of 2 to 10 points, and self-efficacy using one item with a score range of 1 to 5 points.

### Data Collection Procedure

After the student was sampled from the list, he or she was contacted by the first author during break time or evening after clinical practice. After, accepting to participate, a participant found a convenient venue for responding to the questionnaire, filled the informed consent form first and then responded to the questionnaire in the presence of the first author. Then the first author collected the completed questionnaires back.

### Data Analysis

Data were analysed using IBM SPSS Statistics software Version 25. Sample characteristics, including demographic data, knowledge, prevalence of HBV screening and vaccination, and their beliefs per HBM, were analysed using descriptive statistics using frequencies and proportions. Chi-square and *t*-tests were also used to compare subgroups of the sample based on various demographic characteristics, knowledge scores, prevalence, and responses to their beliefs per HBM. The *t*-test was also used to compare the mean score differences on HBM domains among the vaccinated and unvaccinated. Multivariate logistic regression models were used to examine the association between social demographic factors and the domains of the health belief model as predictors of HBV vaccination. The multivariate logistic regression model was first constructed by using sociodemographic variables that showed a significant relationship with HBV uptake in Chi-square analysis as predictors and HBV uptake as a dependent variable. In addition, the unadjusted and adjusted multivariate logistic regression models were performed by using HBM domains as predictors of HBV uptake. The moderation effect of the social demographic variables that significantly predicted the uptake of HBV was included in the adjusted model with the HBM domains. The analyses assumed a 2-tailed distribution, and proportions, *X*^2^ values, *t-statistics,* and adjusted odds ratios (AOR) are reported along with their 95% confidence intervals (CIs), and the results were considered significant with the α level set at 5%.

### Ethical Consideration

This study received ethics approval from the University of Dodoma Research Ethics Committee (MA.84/261/02/235). All participants signed informed consent forms before participating in this study. Participants were treated with respect and dignity, following the ethical principles as prescribed by the Declaration of Helsinki.^28^

## RESULTS

### Sociodemographic Characteristics

All sampled undergraduate clinical nursing students (n = 229) accepted to participate in this study (response rate = 100%). There was an equal proportion among those in the second year of study (33.6%, n = 77), the third year (33.2%, n = 76), and the fourth year (33.2%, n = 76). Majority of the participants (66.8%, n = 153) were male. The overall mean age of participants was 23.8 (SD = 2.7) years. The fresh from school (pre-service) students (n = 216, mean age 23.37 years) were significantly younger than the in-service (joined the degree of nursing after having worked as nurses with a diploma qualification) students (n = 13, mean age 31.23, SD = 6.016); *P* <.001. The majority of participants (94.8%, n = 217) were single. Due to university regulations on health insurance for students, almost all (99.6%, n = 228) participants were covered under the National Health Insurance Fund (NHIF), while only one participant (0.4%) was covered under the Community Health Fund (CHF). Table 1 summarises the demographic characteristics of the participants.

**Table 1.** Participants' Sociodemographic Characteristics

Variable	Frequency / mean	Percent/SD
Sex		
Male	153	66.8
female	76	33.2
Overall age (Mean, SD)	23.82	2.663
Year of study		
2nd year	77	33.6
3rd year	76	33.2
4th year	76	33.2
Marital status		
Single	217	94.8
Married	12	5.2
Type of entry to study program		
In-service	13	5.7
Pre-service	216	94.3
Male guardian's occupation		
Employed/business in health sector	4	1.7
Employed in non-health sector.	58	25.3
Unemployed/business in non-health sector	156	68.1
Female guardian's occupation		
Employed/business in health sector	11	4.8
Employed in non-health sector.	37	16.2
Unemployed/business in non-health sector	170	74.2
Financial support		
HESLB	139	60.7
Parent/guardian	17	7.4
Salary	10	4.4
Both parent and HESLB	63	27.5
Health insurance cover		
NHIF	228	99.6
CHF	1	0.4

Note:

HESLB, Higher Education Students' Loans Board; NHIF, National Health Insurance Fund; CHF, Community Health Fund

### General Knowledge on Hepatitis B Infection and Vaccination

The mean score for general knowledge of HBV infection and vaccination as tested using 23 items was 18.14 (SD = 2.35) with the lowest scoring 5.0 and the highest scoring all 23 points. There was no difference in mean scores between male and female participants (t =.84; *P =.403*), between in-service and pre-service participants (t = 1.23; *P =.219*), or among different categories of fathers' occupations (F (2, 215) =.155, *P =.856*). However, a difference in knowledge was noticed among two classes, where the 3rd year had a significantly higher mean knowledge score compared to the 2nd year (F (2, 215) = 6.652; *P =.001*); with a mean difference of 1.35; 95% CI, 0.45 to 2.24). No difference in knowledge was noticed for 2nd year vs. 4th year and 3rd year vs. 4th year. In addition, those who were vaccinated scored higher in knowledge of Hepatitis B infection and vaccination (mean score (M) = 18.78, SD = 2.07) compared to those who were not vaccinated (M = 17.92, SD = 2.40); t = 2.436, *P =.016*.

### Prevalence of Screening and Vaccination Against HBV and their Predicting Factors

Overall, 32.3%, (n = 74); (95% Confidence Interval (CI, 26.3% to 38.8%) students had ever been screened for HBV. The proportion of females screened were twice as high (48.7%, n = 37) compared to males (24.7%, n = 37); *X*^2^ = 13.936 *P* < .001. In addition, final year students (48.7%, n = 37) made a greater proportion among the screened students compared to 3^rd^ years (26.3%, n = 20) and second years (22.1%, n = 17); *X*^2^ = 14.251 *P* =.001; Also, the in-service students turned up for screening (76.9%, n = 10) more than freshers (29.6%, n = 64); *X*^2^ = 12.539 *P* <.001.

As for vaccination against HBV, the overall prevalence was 25.8%; (95% CI, 20.2% to 31.9%). Females had the proportion of 40.8%, (n = 31) whereas males had 18.3%, (n = 28). Greater proportions of vaccination were seen among females compared to males (*X*^2^ = 13.427, *P*<.001), among the married than singles (*X*^2^ = 11.077, *P* =.001) among in-service than pre-service (*X*^2^ = 13.615, *P* <0.001) and among students who depended on salary to pay for their tuition and living costs at the college (*X*^2^ = 19.175, *P* <0.001). Nevertheless, after a multiple regression analysis of these five (5) variables we found only 3 factors remaining significant; being female (AOR = 2.098, 95% CI, 1.070 to 4.112, *P* =.031), being a 4^th^ year student (AOR = 2.957, 95%CI = 1.261, 6.938, *P* =.013) and score of knowledge on HBV (AOR = 1.171, 95% CI, 1.000 to 1.371, *P* =.049), (Table 2).

**Table 2.** Multiple Logistic Regression of the Sociodemographic Predictors of Vaccination

Variable	AOR	95% CI		p
		Lower	Upper	
Sex				
Male	1			
Female	2.098	1.070	4.112	.031
Year of study				
2nd year	1			
3rd year	1.536	0.632	3.738	.344
4th year	2.957	1.261	6.938	.013
Marital status				
Single	1			
Married	0.639	0.050	8.230	.731
Type of entry to study program				
In-service	1			
Pre-service	0.264	0.018	3.786	.327
Financial support				
HESLB	1			
Parent/guardian	1.958	0.651	5.894	.232
Salary	5.146	0.215	123.401	.312
Both parent and HESLB	1.074	0.507	2.276	.852
Knowledge	1.171	1.000	1.371	.049

Note:

HESLB, Higher Education Students' Loans Board.

### Scores in Domains of Health Belief Model and their Predictive effects on Vaccination

Table 3 shows the mean score differences on HBM domains among the vaccinated and unvaccinated. In the total scores in the belief subdomains of the HBM, only two: perceived benefit (mean difference = 0.491, 95% CI = 0.103–0.879, *P* =.013) and self-efficacy (mean difference = 0.320, 95% CI, 0.084 to 0.555, *P* =.008) showed significant differences between the vaccinated and unvaccinated groups. The scores in the remaining three belief subdomains—perceived threat, perceived barrier, and cues to action—were similar in both the vaccinated and unvaccinated groups. After a multivariate logistic regression model that included HBM domain variables and adjusted for sex, year of study, and HBV knowledge score, those who perceived the benefit of the HBV vaccine had 40% more odds of vaccination uptake compared to those who did not perceive its benefit (AOR = 1.40, 95% CI, 1.05 to 1.86, *P* =.022). In addition, those who had self-efficacy had 87% more odds of vaccination uptake compared to those who perceived an absence of control to take up the complete dose of vaccine (AOR = 1.87, 95%CI, 1.12–3.11; *P* =.016).

**Table 3.** Mean Score Differences on HBM Domains Among Vaccinated and Unvaccinated

HBM domains	Vaccinated group Mean (SD)	Unvaccinated group Mean (SD)	Mean difference (SD)	95% CI of Mean Difference		t-statistic	p
				Lower	Upper		
Perceived Threat (5 items - 25 Points)	20.93 (3.23)	20.72 (3.14)	0.209	-0.73	1.15	0.437	.663
Perceived Benefit (One item – 5 points)	3.93 (1.23)	3.44 (1.32)	0.491	0.103	0.879	2.493	.013
Perceived Barrier (Two items – 10 points)	4.90 (1.36)	4.85 (1.35)	0.045	-0.358	0.449	0.222	.825
Cues to action (Two items – 10 points)	7.75 (2.11)	7.28 (1.91)	0.469	-0.115	1.054	1.582	.115
Self-efficacy (One item – 5 points)	4.66 (0.58)	4.34 (0.85)	0.320	0.084	0.555	2.682	.008

**Table 4.** Multiple Logistic Regression of The HBM Domains as Predictors of Vaccination

Variable	AOR	Model 1† 95% CI		p	AOR	Model 2‡ 95% CI		p
		Lower	Upper			Lower	Upper	
Perceived Threat	0.99	0.89	1.09	.790	0.96	0.86	1.07	.502
Perceived Benefit	1.35	1.04	1.74	.023	1.40	1.05	1.86	.022
Perceived Barrier	1.14	0.89	1.45	.299	1.23	0.95	1.60	.122
Cues to action	1.08	0.91	1.26	.382	1.10	0.93	1.31	.281
Self-efficacy	1.85	1.11	3.09	.018	1.87	1.12	3.11	.016

† Model 1. Only HBM domain variables entered in the model.

‡ Model 2. Variable of model 1 entered and adjusted for sex, year of study and knowledge

## DISCUSSION

This study examined the prevalence, uptake of HBV vaccine and association between nursing students' sociodemographic factors, knowledge of HBV infection and vaccination, domains of the health belief model (HBM), and the uptake of HBV vaccination. The findings revealed a low level of screening as well as vaccine uptake among clinical nursing students, whereas those who had good knowledge about HBV infection and vaccination, females, and final-year students showed a better response to vaccination uptake than their counterparts. As for the belief domains of HBM, the study revealed that those who perceived the benefit of the HBV vaccine and those who had the self-efficacy to take up the complete dose of the vaccine had higher odds of taking up the vaccination.

The fact that a low number of nursing students turned up for screening and vaccination in these settings is not surprising and could be explained beyond the factors that have been identified in this study as associated with HBV vaccination uptake. From this study, we identified that those who were screened for HBV infection were about one-third of the population. This proportion is significantly lower than that ever found in other studies conducted in Africa,^29^ but it reflects the reality of most health care workers and medical and nursing students. Studies conducted in the context of Tanzania, one at the national hospital^13^ and a multi-country study^29^ revealed a high rate of health care workers who do not know their serostatus of HBV infection, where students represented the largest proportion.

As for HBV vaccination uptake, the situation was even worse among students in our study. Only a quarter (25.8%) were vaccinated against HBV. Although other studies in Tanzania^13^, others parts of Africa^29–31^ or in other regions^32^ have shown a low rate of vaccination among nursing and medical students as well as health care workers, the rate of vaccinated clinical students obtained in this study was shockingly low. While the commonest reasons for non-vaccination in those other studies included lack of awareness of the vaccination programme, unavailability of the vaccines, and cost, the reasons might be similar in this study since our findings indicate the most prevalent group for vaccination was the final years and those who have knowledge about HBV infection and vaccination. This may imply that the final year students have more exposure to clinical practice and, therefore, are knowledgeable of the existence of the vaccination programme and have had a chance to vaccinate once the vaccines are available compared to their counterparts.

We also identified that females were more likely to have been vaccinated than males. We cannot conclude from this pattern of findings; however, we hypothesise that this might be attributed to the large proportion of in-service students in the final year who disproportionately tend to be females. The in-service students in this programme have clinical experience and tend to be knowledgeable about clinical practice, as do the risks associated with exposure to HBV, making them more likely to take up the vaccine. As for domains of the health belief model, this study identified that perceived benefit, that is, having perceived that Hepatitis B vaccination would minimise chances of getting infected, as well as self-efficacy, defined as self-confidence in being able to take up the complete dose of vaccine, were associated with 40% and 87% higher chances of vaccination uptake, respectively. Other domains did not show any effect on one's decision to take up the vaccine. Researchers are in a position to determine that each individual construct of the HBM (domain of beliefs) could possibly contribute to influencing action as well as the quantitative relationship between these variables, such as the total perception of benefit minus the total perception of threat.^21,22^ would predict the likelihood of an action. Both the first and second explanations defining the quantitative relationship between perceived benefit and perceived threat to influence their decisions for vaccination may have been the case for this population; however, the narrow difference between the scores in each of the constructs between the vaccinated group and the unvaccinated group may have overshadowed the effect, making perceived threat as an individual construct not show its effect. Nevertheless, perceived benefit as well as subjective norm “self-efficacy” have also been shown by other studies relating to vaccination as strong predictors of intention to vaccinate or minimise vaccine hesitancy.^33,34^

This study also found that the self-efficacy of the clinical nursing students in this setting also predicted nearly twice (87%) higher odds of vaccination compared to those who perceived an absence of control. Self-efficacy was explained by Bandura (1977, 1986, 1997) in a social cognitive theory as “an individual's belief in his or her capacity to execute behaviours necessary to produce specific performance attainments”.^35^ It is a state when an individual perceives to have “ability” to rather than to believe he or she “will” pursue certain goals for an outcome to occur.^35^ Self-efficacy can have a negative effect and influence one's decision to not take a vaccine when they believe they have the ability to prevent the vaccine-preventable disease without a vaccine.^36^ On the contrary, in a similar case as what we found in this study, self-efficacy is linked to a belief that they are able to take the vaccine, manage the pain or side effects that would be associated with the vaccine, and achieve the goal of preventing the disease under which the vaccine is taken.^37–39^ Thus, the findings of this study agree with other studies to suggest that building self-efficacy in the target population can be relevant specific interventions that can improve vaccination uptake, not just hepatitis B but other vaccination programmes such as COVID-19 vaccines.

### Implications

The findings of this study underscore the findings of other studies, which found that the vaccination rate for hepatitis B among clinical nursing students is overall low. This implies that their risk of HBV infection is even higher than the experiences of clinical nurses since clinical nursing students engage in clinical practices much more or less the same as nursing staff without having adequate clinical experiences and thus pose a higher risk of needle sticks and other injuries. ^20^ Deliberate interventions to improve their vaccination uptake must be devised. That could be through extending the campaigns to students living premises or classes to capture their attention when they miss it in the clinical areas; subsidising the cost for clinical students so that they can afford it if they are to pay for vaccination; or selectively availing the vaccines for clinical students through government pay. As for relevant interventions targeting the barriers to HBV vaccination among clinical nursing students, the domains of the health belief model can help to predict the nursing student individuals who are likely to take the vaccines and those who are not. Domain-focused interventions that, for example, target improving one's self-efficacy about their ability to take up the vaccine or proving more knowledge on the benefits of the vaccine would help to improve the vaccination rate. ^20^

### Limitations

Despite the findings of this study putting into context the vaccination status of the clinical nursing students and their influencing factors in Tanzanian settings, the results of this study must be interpreted with caution due to the following limitations: First, given that this was a cross-sectional study, it was beyond our scope to establish causality. Thus, while we could explain the association between perceived benefit and self-efficacy as predicting vaccination uptake and recommend interventions that focus on improving positive perceptions of the beneficial effects of vaccines as well as interventions focusing on improving one's self-efficacy, this relationship could represent a different phenomenon. There could be a chance that one positively supports the benefit of the vaccine after having vaccinated as a coping mechanism from the threats of those who have hesitancy and also proving off the ability to take up the vaccine after having seen the injection, he or she has taken is showing positive results on him. To best capture the relationship between these variables, future studies may need to use a longitudinal approach, such as a prospective cohort study. Secondly, there could be a chance that some students who were unvaccinated identified themselves as vaccinated or vice versa, which could have biassed the results, as the postvaccination ID cards were not used to confirm vaccination status. Nevertheless, we asked the participants to be as honest as possible, as their vaccination status would not influence the privileges they had as students. Thirdly, this study was limited to students at the University of Dodoma. In Tanzania at large, there are many universities that offer a Bachelor of Science in Nursing. There may be a chance that clinical nursing students from other universities exhibit different behaviour patterns relating to HBV vaccination uptake. Future studies should include students from other universities.

## CONCLUSION

This study found that HBV vaccination uptake among undergraduate clinical nursing students is low, much lower than many studies done in Africa and other countries around the world. Sociodemographic factors such as good knowledge about HBV infection and vaccination, being female and being a final-year student, as well as two domains of HBM, including “perceived benefit” of HBV vaccine and “self-efficacy” to take up the complete dose of vaccine, are associated with HBV vaccine uptake. Improving awareness and knowledge about HBV infection and HBV vaccination and building the self-confidence of clinical nursing students may improve vaccination uptake.
